# The Technique and Material Used to Join Transfers Affect the Accuracy and Final Fit of Implant-Supported Prostheses— *In Vitro* Study

**DOI:** 10.1055/s-0044-1779422

**Published:** 2024-05-28

**Authors:** Guilherme de Aguiar Mariotto, Anna Caroliny Detogni, Monica Cavalheiro Persh, Mário Alexandre Coelho Sinhoreti, Veridiana Camilotti, Márcio José Mendonça

**Affiliations:** 1 Department of Restorative Dentistry, State Western University of Parana (UNIOESTE), Dental School, Cascavel, PR, Brazil; 2 Department of Restorative Dentistry, Dental Materials Division, Piracicaba Dental School, University of Campinas (UNICAMP), Piracicaba, SP, Brazil

**Keywords:** Implant-supported prostheses, transfers, dental impression technique, implant components

## Abstract

**Objectives** This study evaluated the linear dimensional change of polymerization of three materials and two techniques of the union of molding transfers for implant-supported prostheses used in the open-tray technique.

**Materials and Methods** A nylon maxilla-shaped matrix was made, two osseous integrated implants were installed, and, over these two, straight conical mini-pillars were installed. Open-tray impression transfers were attached to the mini-pillars, and a silicone guide was made to standardize the connections between the transfers. The samples were divided into six groups ( *n* = 20): PA (Pattern Resin LS, chemically activated acrylic resin in the single step technique); DU (Durallay, chemically activated acrylic resin in the single step technique); BI (Protemp4, bisacrylic resin in the single step technique); PAC (Pattern Resin LS in sectioning and joining of segments technique); DUC (Durallay, in sectioning and joining of segments technique); and BIC (Protemp4, in sectioning and joining of segments technique). The linear dimensional change values that occurred among these transfers were measured in a profile projector (VB300; Starret) coupled to the Quadra Check device, with a resolution of 0.001 mm, performed by a single calibrated operator.

**Statistical Analysis** Data were submitted to a two-way analysis of variance and Tukey's test ( *p* < 0.01).

**Results** Statistically significant mean values were found in all comparisons. The PA showed the lowest mean values (µm) of linear dimensional change, both in the single-step technique and in the sectioning and joining technique, in the following order: BI 255.73 (3.81), DU 173.75 (2.30), PA 95.97 (3.20), BIC 23.82 (1.71), DUC 20.85 (2.53), and PAC 13.27 (2.09). The single-step technique showed the worst results, regardless of the material.

**Conclusion** The sectioning and joining technique reduced the dimensional change in all materials, and the Pattern Resin LS showed the lowest shrinkage mean values, followed by Durallay and Protemp4.

## Introduction

The failure of implant prostheses is due, in part, to the lack of precision and adaptation of the connection system of these prostheses. 1 The precise transfer of implants within the oral cavity is a fundamental requirement for ensuring the satisfactory and passive function of implant-supported partial or complete dentures. 2 This is because the infrastructure and prosthetic components are fabricated based on the working model derived from this impression, which must faithfully replicate the anatomical details. 1 3 4

The absence of passivity between the metallic infrastructure and the implant-supported prosthesis may result in biological problems such as the increasing biofilm accumulation in areas of maladjustment, microfractures of bone tissue, and marginal ischemia zones, in addition to mechanical problems related to the system, such as loosening of fixation screws, fracture of prosthetic components, or even fracture of the implant itself. 2 5 6 7 According to a previous study, variables such as the type of connection between implants, copings or transfers, number of implants, angulations, materials, and impression techniques used in the transfer step can influence the quality of this transfer. 2 8 9 10 Hence, the accuracy of impression materials as well as the impression technique related is being widely studied, due to their direct influence on the quality and longevity of the rehabilitative treatment performed. 3

Different techniques are proposed for the transfer step among which direct impression techniques with an open tray or indirect impression with a closed tray are the most common. 9 11 The open-tray technique is the most recommended approach since it allows obtaining impressions and transfers accurately regardless of the material used. 9 11 In this technique, the transferees have a rounded or conical format and are not attached to the mold; however, they can be reinserted into this mold prior to filling with dental stone to obtain the working model. 11 Concerning the closed-tray technique, this is less used then the first due to the greater difficulty in obtaining precision between the positioning of the transfers and the impression material. 9

For impression accuracy improvement, the fixation of transfers through splinting can be performed to avoid the unitary movement of these components in situations of multiple components. 9 12 Splinting the transfer' junction with self-curing acrylic resins is the best technique for this purpose because it presents a greater precision when compared with the absence of bonding, despite their high polymerization shrinkage. 9 13 Among the materials evaluated for this purpose, acrylic resins Durallay and Pattern Resin LS are the most used. 14 15 16 17 18 19 In addition, the use of bisacrylic resins for this splinting have shown satisfactory success, besides greater ease of handling, due to application through self-mixing tips, precision comparable to self-polymerizable acrylic resins, and high flexural strength. 14 Furthermore, a braided dental floss among the transfers associated with light-curing resin composites and/or flowable resin composites also can be used for this purpose, to avoid a possible displacement of the components from their original position, that can occur due to the polymerization shrinkage of self-curing acrylic resins, when these are used for bonding. 12 20 21

Despite the influence of the polymerization shrinkage of the materials used for joining the components during the transfer step on the final performance of the prosthesis, few studies have evaluated the dimensional change caused by the polymerization shrinkage of the different materials that can be used for this approach. Thus, this study aimed to comparatively evaluate the linear dimensional changing of three materials associated with two different join techniques between the transfers, these being two self-curing acrylic resins and a bisacrylic resin. The null hypothesis was that there were no statistically significant differences between the materials and techniques used.

## Materials and Methods

### Sample Size Calculation

The sample size calculation was done based on F family probability distributions, with repeated family design, with interaction within and between factors. The effect size used was 0.15, type 1(α) error of 0.01, and analysis power of 0.95 ensuring a minimum of 84 sample units (test specimens). For convenience, 120 specimens were made, with 20 specimens per group (6 groups in total). The sample calculation was performed using the GPower program (version 3.1.9.2 - University of Düsseldorf, Düsseldorf, Germany).

### Standard Matrix

**Fig. 1:**
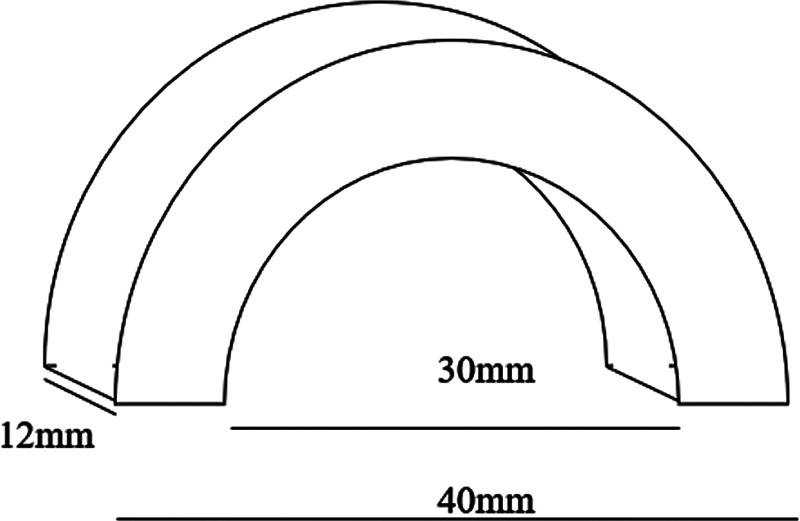
Technical drawing of the nylon matrix for drilling.

A nylon standard matrix was made with the aim of simulating the maxilla. The nylon was used due to its modulus of elasticity similar to that of the medullary human bone. 15 This matrix was made by milling around (BV20, Leetools, Santo André, SP, Brazil) with a nylon 6.0 cylinder (NY6, MGS Plásticos, Pinhais, PR, Brazil) of 50 mm in diameter, with a “C” shape, internal diameter of 30 mm (equivalent to the palatal wall of the maxilla) and external of 40 mm (equivalent to the buccal wall of the maxilla), and height of 12 mm ( Fig. 1 ).

On this matrix, the drilling sequence recommended by the manufacturer of the implants was performed using a bench drill (FBV16, Vonder, Curitiba, PR, Brazil) to ensure better parallelism between the perforations. The entire milling sequence was performed at a speed of 1100 rpm. The implant (Titamax Cortical External Hexagon; Neodent, Straumann, Basel, Switzerland) measuring 3.75 mm in diameter and 11 mm in length, with a 4.1 mm prosthetic platform, was installed with a ratchet-torque meter (Neodent, Straumann, Basel, Switzerland) with a final torque of 45 N/cm ^2^ .

**Fig. 2:**
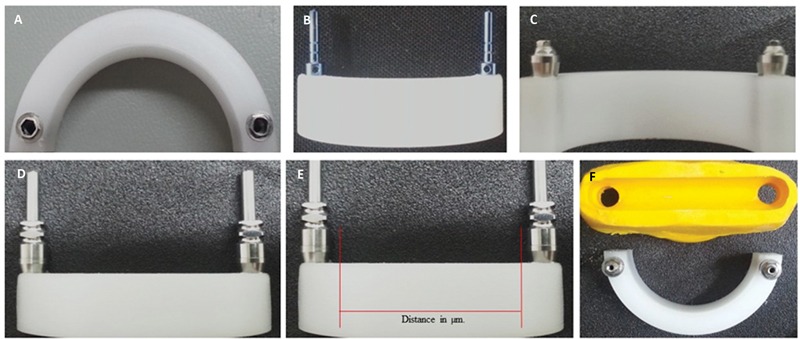
Implant and matrix positioning steps. ( **A** ) Position of the implants in the matrix—top view; ( **B** ) Position of the implants in the matrix—anterosuperior view; ( **C** ) Installation of mini pillars; ( **D** ) Impression transfers installed; ( **E** ) Measurement of the distance between the transfers in the matrix; ( **F** ) Finished addition silicone guide.

The reaming was performed in the areas of the matrix corresponding to the positions of the upper second premolars ( Fig. 2A ), as this is the position with the greatest distance on a protocol bar. The implants were positioned parallel to each other and perpendicular to the matrix ( Fig. 2B ). The implant platforms were positioned at the same level as the upper surface of the matrix, similar to a bone-level installation. Mini-pillar-type prosthetic components (Straight Slimfit, Neodent, Straumann, Basel, Switzerland) were installed on the implants with a transmucosal height of 4 mm ( Fig. 2C ). The mini-pillars received the recommended torque of 32 N/cm ^2^ with the manufacturer's torque meter ratchet. Impression transfers (Mini-abutment Open Tray Transfer, Neodent, Straumann, Basel, Switzerland) were installed on the mini-abutments with a bi-digital driver recommended by the manufacturer ( Fig. 2D ).

The distances between the closest portions of these transfers were measured ( Fig. 2E ) using a vertical bench profile projector (VB300; Starrett, Athol, Massachusetts, United States) coupled to the Quadra Check device, with a measurement resolution of 0.001 mm. Ten measurements were performed, and the arithmetic mean value was considered the initial standard value. Then, the union between the impression transfers was performed with a plastic straw of 5 mm in diameter fitted between the centers of the transfers with wax for prosthetic sculpture (Cera PK, Kota, Cotia, SP, Brazil). The straw was fixed with wax embracing the entire diameter of the two transfers. After the set was fixed, an addition silicone guide was made by adding the putty soft normal set consistency (Elite HD+ Putty Soft, Zhermack, Badia, Italy) to create the guide to pour the different bonding materials of the transfers with the same dimensions. This guide was cut in its upper region with a bench plaster cutter (VH, Essence Dental, Araraquara, SP, Brazil), at the limit of the upper wall of the plastic tube, to obtain a guide height of 5 mm.

After cutting, the upper edges were removed with a metal drill (5 mm high-speed steel; Vonder, Curitiba, PR, Brazil) on a bench drill (FBV16, Vonder, Curitiba, PR, Brazil). The objective was to create parallel sidewalls to avoid sample retention after the complete set. This additional silicone guide was used to produce specimens with the same dimensions for all materials ( Fig. 2F ).

### Union of Transfers

**Fig. 3:**
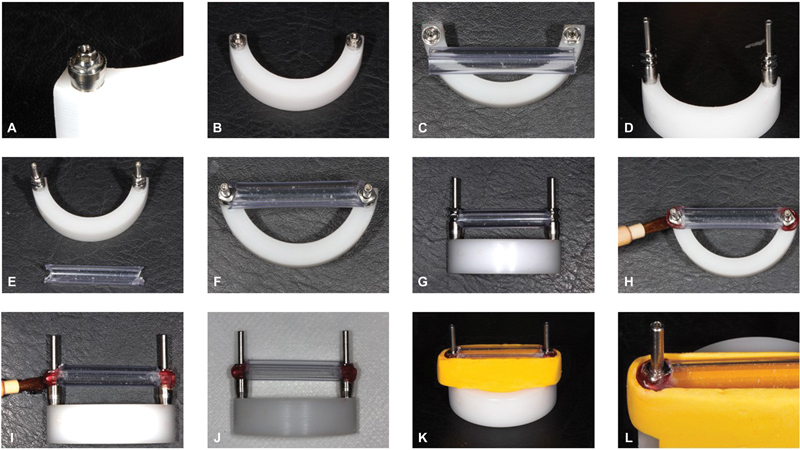
Construction of the silicone guide: ( **A** ) right mini-pillar installed; ( **B** ) mini-pillars installed; ( **C** ) measurement of the PVC straw for cutting; ( **D** ) installation of open tray transfers on the mini-abutments; ( **E** ) cutting out the PVC straw; ( **F** ) test of the PVC straw – occlusal view; ( **G** ) test of the PVC straw – anterior view; ( **H** ) joining the PVC straw to the impression transfer – occlusal view; ( **I** ) joining the PVC straw to the molding transfer – front view; ( **J** ) straw attached to the molding transfers; ( **K** ) application of addition silicone under the straw attached to the transfers – anterior view; ( **L** ) application of addition silicone under the straw attached to the transfers – occlusal view.

After making the guide, the transfers were joined with different materials and techniques, and the groups were separated: PA, chemically activated acrylic resin in the single-step technique (Pattern Resin LS, GC America Inc, Alsip, Illinois, United States); DU, chemically activated acrylic resin in the single step technique (Durallay, Reliance Dental, Worth, Illinois, United States); BI, bisacrylic resin in the single-step technique (Protemp4, 3M Oral Care, St. Paul, Minnesota, United States); PAC, Pattern Resin LS acrylic resin in sectioning and joining of segments technique; DUC, Durallay acrylic resin in sectioning and joining of segments technique; and BIC, Protemp4, bisacrylic resin in sectioning and joining of segments technique ( Fig. 3 ). As a total, 120 specimens were made ( *n* = 20).

The sample units were considered the two impression transfers joined with the respective bonding materials, after their removal from the silicone guide. The preparation of the specimens was performed in an environment with a controlled temperature of 22 ± 1°C. In the groups in which acrylic resin (PA, PAC, DU, DUC groups) was used, the powder-liquid agglutination technique was used according to the manufacturer's recommendations. The manipulation of the material was performed with the aid of a brush, used to dispense the powder portions (Kit Pattern Resin LS, GC America Inc, Alsip, Illinois, United States). Initially, the regions around the transfers were filled in on both sides and then the rest of the guide was completed until the entire segment was joined. After a period of 5 minutes from the addition of the last portion of the material, the initial polymerization was considered completed for all evaluated materials.

**Fig. 4:**
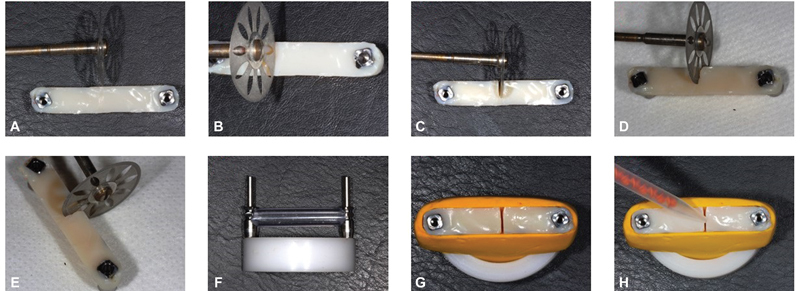
Bar section and re-joining: ( **A** ) bar and cutting disc; ( **B** ) disc positioned for cutting; ( **C** ) start of cutting; ( **D** ) bar cutting; ( **E** ) bar cutting; ( **F** ) straw attached to the molding transfers; ( **G** ) installation of the cut segments on the guide; ( **H** ) positioning of the mixing tip to inject the joining material.

In the groups in which a Protemp4 bisacrylic resin was used (BI and BIC groups), it was injected through a mixing tip recommended by the manufacturer (Garant mixing tips, 3M Oral Care, St. Paul, Minnesota, United States) along the entire length of the silicone guide, and removal was performed after the complete polymerization of the material ( Fig. 4 ).

**Fig. 5:**
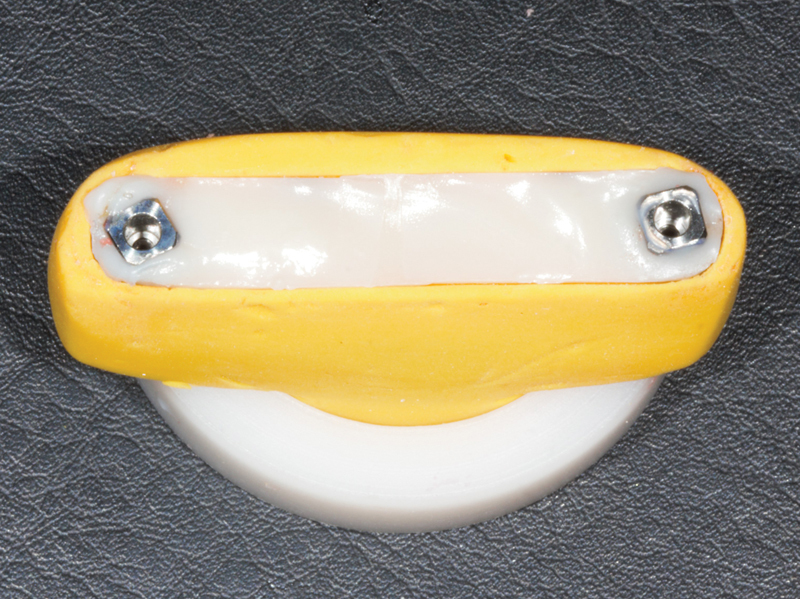
Union of segments.

For the PUC, DUC, and BIC groups, after the initial polymerization of 5 minutes, the bar was removed from the guide and sectioned using a segmented double-sided diamond disc (0.10 × 22 mm; KG Sorensen, Barueri, SP, Brazil), coupled to a micromotor and straight piece (500; Kavo do Brasil, Joinville, SC, Brazil). To standardize the section, the specimens were fixed in a type IV dental stone pattern (Herodent, Coltene, Rio de Janeiro, RJ, Brazil) with mini-pillar analogs, leaving the bar parallel to the ground, and the diamond disc was moved transversely to the bar, perpendicular the same, obtaining two segments of equivalent lengths. The sectioned parts were repositioned on the silicone guide and joined with the same material and technique previously described ( Fig. 4 ). The final appearance of the sample is seen in Fig. 5 . After a period of 5 minutes of polymerization of the union of the segments, the samples were stored at a controlled temperature of 21°C until the measurements.

After the final setting, each sample received its identification, and the time of completion of preparation and reading of each specimen was noted, the standard reading time being 120 ± 5 minutes after preparation. The purpose of this procedure was to reproduce the average interval time between the union of the transfers in the mouth, molding, finalization of procedures with the patient, the time that the laboratory would take to collect the work in the office and reach the place of production of dental stone casts. Each specimen was measured three times, and the arithmetic mean between these was considered.

The specimens were measured in a vertical bench profile projector (VB300, Starrett, Athol, Massachusetts, United States) respecting the same time between the completion of each sample and the measurement. Thus, all were measured after the same time of preparation had elapsed. As in the initial standard matrix, the distance between the closest points of the two impression transfers of each specimen was measured, after its polymerization and removal of the silicone guide. The mean of each specimen was subtracted from the initial standard mean value of the matrix, thus obtaining the mean value of dimensional change (positive value indicates contraction and negative value, expansion). All procedures for making and measuring the specimens were performed by a single operator, previously calibrated.

### Statistical Analysis

Statistical analysis was performed using the Bioestat software (Bioestat 5.3; Instituto Mamirauá, Tefe, Amazonas, Brazil). The data obtained were initially submitted to the test of adherence to the normality curve using the D'Agostino-Pearson test. Then, two-way analysis of variance was used (α = 0.01), followed by Tukey's posthoc test to perform multiple comparisons between groups (α = 0.01).

## Results

The results obtained for the factorial analysis of variance showed statistically significant differences for both evaluated factors: bonding material and technique used. Significant differences were also found in the interaction between these factors. The mean values of linear dimensional change of the assessed groups are shown in Table 1 . The analysis of Table 1 shows that all experimental groups had statistically significant differences among themselves, regardless of the assessed factor: bonding material or technique. Mean percentages of linear change were as follows: Group BI 0.86%; DU 0.58%; PA 0.32%; BIC 0.08; DUC 0.07%; CAP 0.04%.

**Table 1:** Mean (µm) and standard deviation (± ) of linear dimensional change in the evaluated groups

Bonding material and technique	Protemp4	Durallay	Pattern
Single-step	255.73 ± 0.81 Aa	173.7 ± 2.30 Ab	95.97 ± 3.20 Ac
Section and union	23.82 ± 1.71 Ba	20.85 ± 2.53 Bb	13.27 ± 2.09 Bc

Different letters represent statistically significant differences. Lowercase refers to rows (technique used) and uppercase refers to columns (material used) ( *p* < 0.01).

**Fig. 6:**
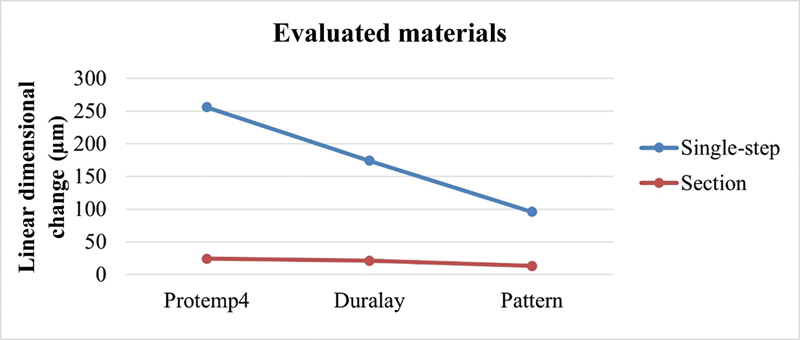
Comparison of the evaluated materials according to the joining technique.

The bonding material factor evaluated using the same technique showed that the Pattern Resin LS resin presented the smallest linear dimensional change, followed by the Durallay resin. Protemp4 bisacrylic resin was the material that demonstrated the highest mean value of linear dimensional change. The positions remained, regardless of which technique was considered ( Fig. 6 ).

**Fig. 7:**
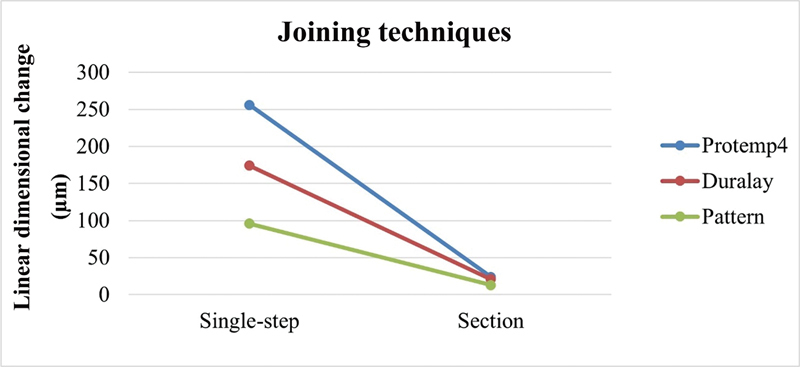
Comparison of evaluated techniques according to the materials used.

When the technique factor was evaluated, statistically significant differences were observed between the techniques for all materials. The lowest polymerization shrinkage occurred in the groups that used the section and union technique compared with the single-step technique ( Fig. 7 ).

## Discussion

In oral rehabilitation using implants, the molding technique with an open tray associated with the union between the transfers is the one that obtains the best results, according to previous studies. 11 13 15 16 17 18 19 In this technique, dental floss is braided between the transfers using the brush technique, when the brush is wetted in the monomer, and then agglutinating the acrylic powder. Then, the acrylic resin is inserted enveloping the transfers completely, to incorporate and unite them. 15 16

This study comparatively evaluated the linear dimensional alteration of three materials with two different bonding techniques between the transfers frequently used in the molding technique with an open tray for implant rehabilitation. The results showed that Protemp4 bisacrylic resin significantly showed the highest mean values of linear dimensional change, followed by Durallay and Pattern Resin LS. Thus, the null hypothesis, that there would be no statistically significant differences between all materials and techniques, was rejected.

The observed differences in linear dimensional change might be attributed to variations in material composition. Pattern Resin LS and Durallay are both acrylic resins containing polymethyl methacrylate. 22 23 However, Pattern Resin LS has the dimethyl-p-toluidine tertiary amine in its matrix, which acts by promoting an increase in the number of linear and three-dimensional bridges of the polymeric chains, thus forming a greater number of branched chains that, consequently, increase the mechanical rigidity of the material and decrease its polymerization shrinkage. 22 This finding corroborates the findings of Gibbs et al 2014, 24 who reported significantly lower polymerization shrinkage values for conventional acrylic resins when compared with acrylic gel resins. 24 In addition, the low degree of linear dimensional change of these materials compared with Protemp4 bisacrylic resin may have occurred because of the transfer agents used, which may have contained the rate of polymerization shrinkage of the materials used. Therefore, the way materials behave when prevented from contracting freely is different from the pure shrinkage of the material.

Regarding the Protemp4 bisacrylic resin used in this study, the results showed that when used in a single step, this material presented the greatest linear dimensional change among the others, making its use unfeasible through this technique. This material contains bifunctional methacrylates, silicon dioxide particles, vinyl copolymers, inorganic filler particles, and bifunctional esters that give the formed polymer structure greater crosslinking, high mechanical resistance, and hardness to the material. 25 Furthermore, this resin has a hydrophobic behavior, ensuring minimal water absorption and satisfactory wear resistance, since its bisacrylic monomers have a rigid central structure that reduces the resin matrix hydrolysis during immersion in saliva. 15 26 These characteristics allow greater flexural strength, hardness, degree of conversion, polymerization shrinkage, and consequently greater linear dimensional change due to the lower molecular weight of the monomers present in its composition compared with the other evaluated acrylic resins.

Concerning the techniques used in association with the different materials, statistically significant differences were observed between all groups, with the sectioning and union technique being the one that best demonstrated to reduce the linear dimensional alteration of the materials used to join the transfers, regardless of the material used. The simple maneuver of cutting and joining the segments promoted the results of all materials similar to the best obtained in the study, with a difference between the best and worst result of 10.55 µm. Therefore, a more precise technique, in theory, could be able to compensate for the high linear dimensional change and justify the use of some materials due to the improvement obtained in the impression. Furthermore, when considering 100 µm as an acceptable misfit, the sectioning and bond technique allowed the three materials used in this study acceptable. 5 27 Besides, assuming that there is no failure in any of the fabrication and transfer steps, only Pattern Resin LS would be within the acceptable shrinkage limit for the single-step technique, with 95.97 (± 3.20) µm.

The clinical implications of these findings should be considered. The linear dimensional changes may impact the fit and long-term success of implant-supported restorations. 28 Future research could explore alternative materials or techniques to minimize linear dimensional changes and enhance the accuracy of impressions. Additionally, investigating modifications or improvements to the bonding techniques may further optimize the fabrication process. In conclusion, the choice of materials and bonding techniques significantly influenced the linear dimensional alteration in implant rehabilitation. Selecting appropriate materials and employing precise bonding techniques are crucial for achieving accurate impressions and ensuring the success of implant-supported restorations. Further studies are warranted to refine existing techniques and explore novel approaches in this field.

## Conclusion

The sectioning and joining technique tested in this study reduced the dimensional change in all materials. Pattern Resin LS showed the lowest shrinkage mean values followed by Durallay and Protemp4.
